# The importance of nickel oxyhydroxide deprotonation on its activity towards electrochemical water oxidation†
†Electronic supplementary information (ESI) available. See DOI: 10.1039/c5sc04486c


**DOI:** 10.1039/c5sc04486c

**Published:** 2016-01-05

**Authors:** Oscar Diaz-Morales, David Ferrus-Suspedra, Marc T. M. Koper

**Affiliations:** a Leiden Institute of Chemistry , Leiden University , PO Box 9502 , 2300 RA Leiden , The Netherlands . Email: m.koper@lic.leidenuniv.nl

## Abstract

Nickel oxyhydroxide (NiOOH) is extensively used for energy storage and it is a very promising catalyst for the oxygen evolution reaction (OER). However, the processes occurring on the NiOOH surface during charge accumulation and OER are not well understood. This work presents an *in situ* Surface Enhanced Raman Spectroscopy (SERS) study of the pH dependent interfacial changes of the NiOOH catalyst under the working conditions used for OER. We demonstrate the important effect of the electrolyte pH on the degree of surface deprotonation of NiOOH, which crucially affects its OER activity. Our results show that the deprotonation of NiOOH produces negatively charged (or proton-deficient) surface species, which are responsible for the enhanced OER activity of NiOOH in highly alkaline pH. Moreover, we provide spectroscopic evidence obtained in an ^18^O-labeled electrolyte that allows us to assign this surface species to a superoxo-type species (Ni–OO^–^). Furthermore, we propose a mechanism for the OER on NiOOH which is consistent with the observed pH-sensitivity, and that also explains why NiOOH is not a suitable catalyst for applications in neutral or moderately alkaline pH (in the range 7–11), apart from the lower stability of the catalyst under these conditions.

## Abstract 2

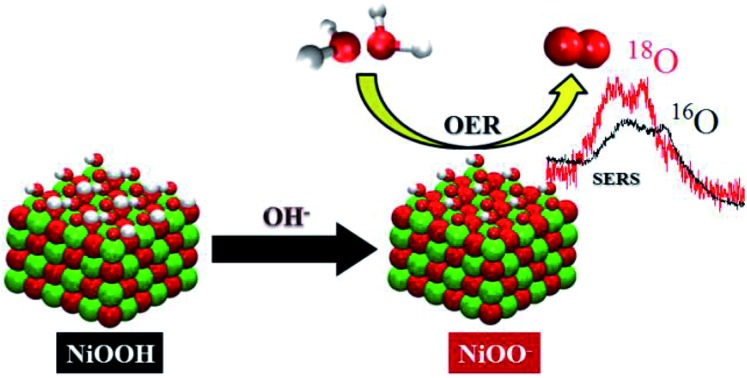
The OER activity of NiOOH enhances at pH > 11 due to formation of superoxo-like species on its surface.

## Introduction

Nickel-based oxides are extensively used for secondary batteries and super capacitors.^1^–^3^ These materials are also very promising catalysts for the OER,^4^–^10^ which is one of the major bottlenecks for solar energy conversion into storable fuels.^11^,^12^ However, the mechanism of nickel charging and its activation towards OER are still a matter of debate. The Bode scheme is one of the accepted mechanisms for the charge/discharge process of the nickel (hydr)oxide, according to which the freshly prepared α-Ni(OH)_2_ oxidizes to form γ-NiOOH;^13^ these phases convert into the more crystalline β-Ni(OH)_2_/β-NiOOH phases upon (electro)chemical ageing. It has been proposed that the formal oxidation state of nickel in the γ-NiOOH lies in the range 3.5–3.67,^9^,^14^ which suggests that some nickel sites in this compound have NiO_2_-like character that may be seen as tetravalent nickel sites; this hypothesis has been supported with X-ray adsorption spectroscopy (XAS), by matching the position of the Ni K-edge of the γ-NiOOH samples with the K-edge of reference compounds in which nickel was thought to be in the Ni^IV^ state (BaNiO_3_ or KNiIO_6_).^15^–^17^ However, the values reported for the oxidation state of nickel in those reference compounds did not consider the possibility of oxygen vacancies, which affect the formal valence of nickel in the compounds and make the conclusions derived from the XAS data uncertain.^18^,^19^

The OER mechanism on a nickel-based catalyst (nickel-borate) was recently studied by Nocera *et al.*,^20^ and they proposed that the formation of the catalytically active species for the OER occurs *via* an oxidative deprotonation of a nickel oxyhydroxide-like structure; the NiOOH proposed by them is dispersed in a polymeric hydrous network similar to the one suggested by Lyons *et al.*^9^,^21^ The charging mechanism of Ni(OH)_2_ in KOH and its activation towards OER was also studied by Merrill *et al.*^22^ by means of Surface Enhanced Raman Spectroscopy (SERS), who reported the appearance of a broad peak in the 900–1100 cm^–1^ wavenumber region when α-Ni(OH)_2_ oxidizes to form γ-NiOOH. This broad feature was attributed to “active oxygen O^0^” within the NiOOH structure. The spectroelectrochemical evidence for the active oxygen species within the oxyhydroxide network raises the question whether this feature may be related to the deprotonated species reported by Nocera *et al.* for the OER active form of nickel-borate catalyst, which heralds the onset of oxygen evolution.

The oxidative deprotonation process to generate the catalytic species for the OER is not particular for nickel. It has been reported that cobalt, iron and manganese-based catalysts also deprotonate prior to oxygen evolution, in processes that are strongly pH-dependent.^23^–^25^ Since the OER activity of NiOOH is also known to be pH-dependent and favored in more alkaline media,^9^,^21^ the appearance of the SERS feature attributed to the “active oxygen” should also correlate with the pH, if this species is related to the formation of OER catalytically active sites in the structure of NiOOH. Following this hypothesis, we present here a systematic *in situ* SERS study of the pH dependence of the catalytic activity of NiOOH towards electrochemical O_2_ generation. Our electrodes consist of NiOOH electrodeposited on gold in a rigorously Fe-free electrolyte; the importance of removing such impurities was recently demonstrated by the Boettcher group.^26^ The elimination of the Fe impurities from the electrolyte allows us to conclusively rationalize pH-dependent activity changes to observed spectral changes in the γ-NiOOH catalyst. Based on these results, we will suggest a mechanism for OER reaction on first-row transition-metal oxides that we believe will be useful for guiding future first-principles calculations of novel catalysts.

## Experimental section

All glassware was rigorously cleaned before starting experiments by boiling in concentrated H_2_SO_4_ to remove metals and organic contaminations, and was subsequently boiled five times in Millipore Milli-Q water (resistivity > 18.2 MΩ cm), which was also used to prepare the solutions for the electrochemical experiments.

The chemicals used in this work were of ultra-high purity: Ni(NO_3_)_2_·6H_2_O (Aldrich trace metal basis, 99.999%), HClO_4_ (Aldrich TraceSelect® for trace analysis, 67–72%) and iron-free NaOH. The purification of commercial NaOH followed the procedure reported by Boettcher's group,^26^ by shaking a 1 M solution of NaOH (30% solution in H_2_O, TraceSelect® for trace analysis) with Ni(OH)_2_ that was precipitated from the 99.999% Ni(NO_3_)_2_·6H_2_O salt. The NaClO_4_ used as supporting electrolyte was prepared by neutralizing the Fe-free NaOH solution with HClO_4_, to minimize the amount of iron impurities present in the solution. The pH of the solutions used in all the experiments of this work was adjusted with HClO_4_, and verified with a pH-meter. All experiments were performed at constant ionic strength, which was kept constant at 0.1 M by adding NaClO_4_ as supporting electrolyte except for the electrolyte at pH 13 and 14, which did not contain NaClO_4_; they were NaOH 0.1 M and 1 M, respectively.

*In situ* Surface Enhanced Raman Spectroscopy (SERS) was performed with a confocal Raman microscope (LabRam HR, Horiba Yobin Yvon) with a 50× objective. The excitation source used was a 30 mW He/Ne laser (633 nm). Backscattered light was filtered with an edge filter at 633 nm, subsequently directed to the spectrograph and to the CCD detector; further details of the setup can be found in ref. 27 and 28. The experiments were made in a two-compartment and three-electrode cell made of glass, with a quartz window at the bottom. A gold spiral was used as counter electrode, Ag/AgCl (sat. KCl) as reference electrode, and nickel electroplated on a roughened gold disk as working electrode; the reference electrode was separated from the working electrode compartment to avoid chloride contamination. The electrochemical experiments were controlled by a μAutolab type III potentiostat/galvanostat (Metrohm-Autolab). Dissolved oxygen in solutions was removed prior to measurements by purging with argon (purity grade 5.0) for at least 30 min, and the argon was kept flowing above the solution during the experiments.

The working electrode used in this work was a gold disk back-contacted with a gold wire and it was not mounted in any material to allow annealing during the cleaning procedure; the electrochemical measurements were performed with the disk in meniscus configuration. Prior to each measurement, the disk was mechanically polished to mirror finish using aqueous diamond pastes (Buehler Limited) with different grain sizes to 0.25 μm, rinsed with Milli-Q water and ultrasonicated during 5 min to remove all residuals of mechanical polishing; next the gold electrode was annealed with a butane flame and electrochemically roughened by 25 oxidation–reduction cycles (ORC) in a 0.1 M solution of KCl. The ORC were performed between –0.30 and 1.20 V *vs.* SCE, during which the potential was held for 30 seconds at the negative limit and for 1.3 seconds at the positive limit; this method has been reported to give a brownish surface that is SERS active.^29^ The roughened gold electrode was thoroughly rinsed with water to measure a cyclic voltammetry in the potential range 0–1.75 V *vs.* RHE in 0.1 M HClO_4_ at 0.05 V s^–1^. The real surface area of the electrode was measured from the charge of the reduction peak of the gold oxide, assuming 390 μC cm^–2^ for the charge for one monolayer of gold oxide.^30^ The surface area obtained from this measurement was used to calculate the current density in the cyclic voltammetry reported in the work. The capacitance-corrected plots of catalytic activity were obtained from the cyclic voltammetry curves by averaging the current of the backward and forward scans.^31^,^32^

Nickel was plated on the roughened gold electrode by galvanostatic electrodeposition from a 5 × 10^–3^ M Ni(NO_3_)_2_·6H_2_O solution, using 0.1 M NaClO_4_ as supporting electrolyte. The deposition was carried out by applying a cathodic current (10 μA) for a given time, in order to obtain *ca.* five monolayers of coverage; the time for nickel plating was calculated according to the real surface area of the working electrode in order to deposit 5 × 726 μC cm^–2^, the latter value corresponding to the charge needed to deposit one monolayer of closely packed metallic nickel from a Ni^II^ solution, taking the atomic radius of Ni as 0.124 nm and its density as 8.908 g cm^–3^.^33^

All potentials in this work are reported *versus* the reversible hydrogen electrode (RHE) in the working pH, unless otherwise stated. The potentials were converted into the RHE scale according to the eqn (1).

1 *E*_RHE_ = *E*_Ag/AgCl(sat. KCl)_ + *E*0Ag/AgCl(sat. KCl) + 0.059ΔpH

where *E*_RHE_ is the potential on the RHE scale, *E*_Ag/AgCl(sat. KCl)_ is the potential applied experimentally and *E*0Ag/AgCl(sat. KCl) is the standard potential of the Ag/AgCl redox couple (in a solution saturated with KCl) on the normal hydrogen electrode scale (0.197 V),^34^ ΔpH accounts for the difference in pH of the working solution with respect to the conditions used for the normal hydrogen electrode (pH = 0). Eqn (1) was verified by measuring the equilibrium potential of platinum in a solution NaOH 0.1 M (pH 13) saturated with H_2_.

The Raman experiments in H_2_^18^O (98% isotopic purity, GMP standard, purchased from CMR) were performed at pH 13 (NaOH 0.1 M). The electrolyte for these experiments was used without further purification, and the electrochemical cell had a smaller internal volume (*ca.* 1 mL); schematic details of this cell can be found in ref. 28.

## Results and discussion

**Fig. 1:**
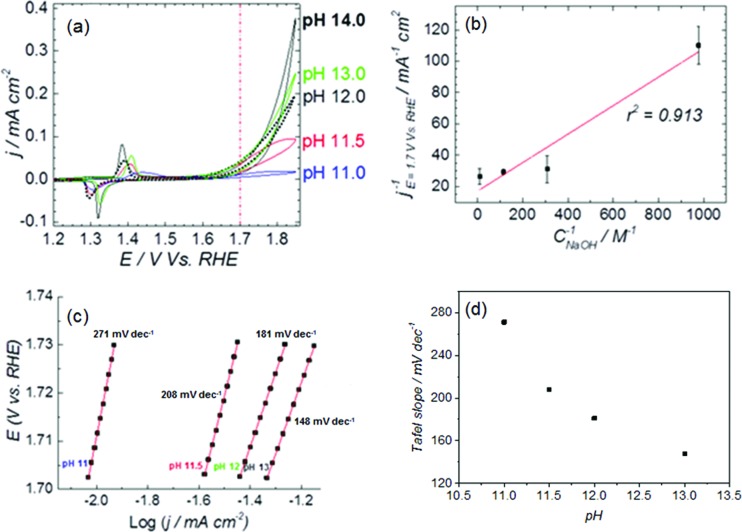
(a) CVs of NiOOH deposited on Au, showing the Ni^II^/Ni^III^ redox peaks and the OER activity at *E* > 1.65 V. Measurements at pH 11–13 were performed at constant ionic strength, adjusted to 0.1 M with NaClO_4_ except for pH 13 and pH 14, those solutions solution were NaOH 0.1 M and 1 M, respectively. Scan rate: 0.01 V s^–1^. (b) Langmuir-type plot of the OER activity as a function of the concentration of NaOH in the electrolyte (pH 11–13), the activity was measured from the CV's as the average of the backwards and forward current density at 1.7 V *vs.* RHE (red dashed line in (a)). (c) Tafel plot, obtained from the CV's in (a) as the average of the backwards and forward current density in the potential region 1.702–1.73 V *vs.* RHE. (d) Tafel slope as a function of the electrolyte pH.

The cyclic voltammetry (CV) in Fig. 1a shows the Ni(OH)_2_/NiOOH (Ni^II^/Ni^III^) redox transition in the potential region 1.3–1.5 V *vs.* RHE. The potential at which the redox transition occurs does not show significant pH dependence (see Fig. S1a and b in the ESI†). However, the OER activity (expressed as current density) does depend on the pH of the electrolyte, as confirmed in the capacitance-corrected plot of OER activity as function of the applied potential in Fig. S2 in the ESI.† Fig. 1a show that the OER activity at pH 11.0 is negligible and increases with the electrolyte pH, with a tendency to saturate at the highest pH values (see Fig. S3 in the ESI†).

The potential of the Ni^II^/Ni^III^ transition and the OER current of NiOOH in 1 M NaOH (see Fig. 1a) compares well with the results reported by Boettcher's group,^26^ and confirms that the hydroxide solution was free of iron traces. We can therefore assert that our NiOOH catalyst is not contaminated with Fe during the electrochemical experiments, and the pH effect is not an artifact caused by impurities in the electrolyte. The elimination of Fe impurities in the electrolyte is important because the presence of Fe in the electrolyte shifts the OER onset potential to lower values due to the formation of NiFe mixed oxyhydroxide (Ni_1–*x*_Fe_*x*_OOH), which has a higher catalytic activity for oxygen evolution than NiOOH itself;^26^ the formation of Ni_1–*x*_Fe_*x*_OOH also affects the position of the Ni^II^/Ni^III^ redox transition, and produces an apparent pH dependence of the redox pair (compare Fig. S1c and d to Fig. S1a and b†). Fig. S3a and b in the ESI† shows polarization curves of Ni(OH)_2_ deposited on Au, obtained in purified (Fe-free) and non-purified electrolytes, respectively. The cyclic voltammetry in the Fe-containing electrolytes (Fig. S3b in the ESI†) differs from the one obtained in Fe-free electrolyte: the Ni^II^/Ni^III^ redox peaks of NiOOH shift with pH and the OER activity increases *ca.* 20-fold from pH 11 to pH 13 whereas the enhancement is about 10-fold for the purified electrolyte. In general, the activity measured in the Fe-containing electrolyte is *ca.* 10-fold higher than in the Fe-free electrolyte.

**Fig. 2:**
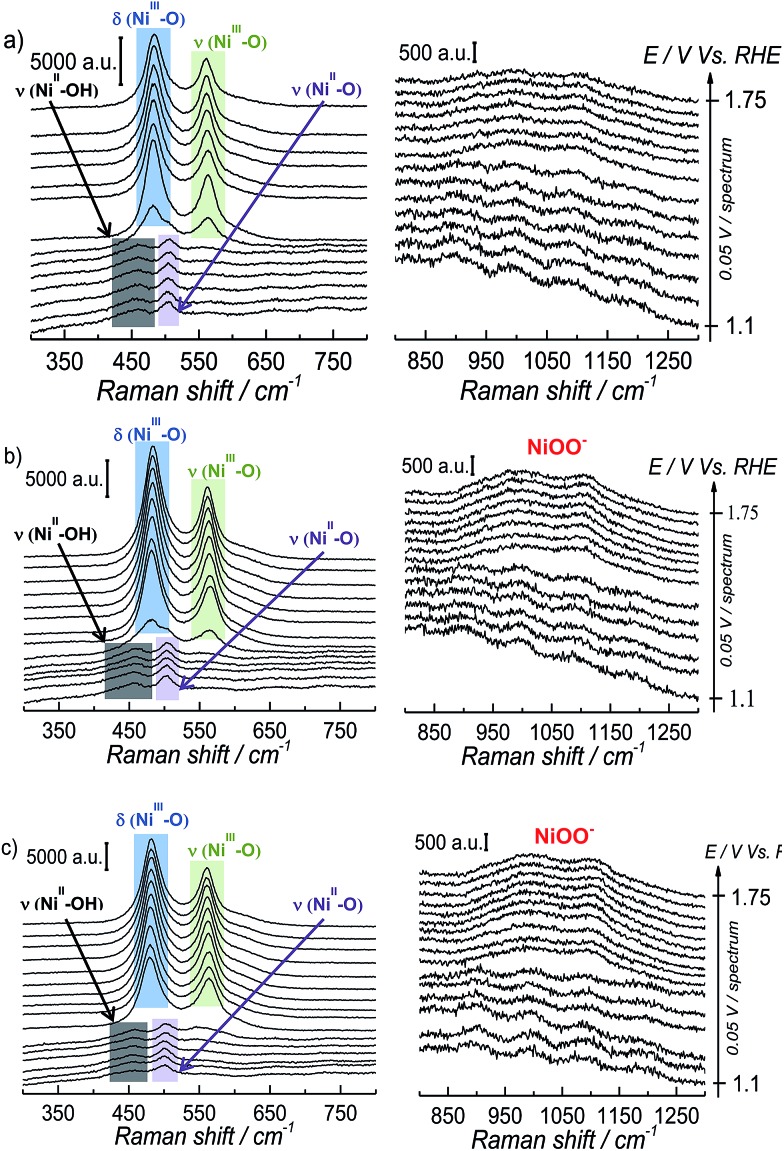
SER spectra obtained at constant potential during the electrochemical oxidation of Ni(OH)_2_ and the subsequent OER on NiOOH at different pH's. The ionic strength of the solution was fixed to 0.1 M with NaClO_4_ except for pH 13, that solution is NaOH 0.1 M. The left panel presents the spectra in the wavenumber region 300–800 cm^–1^ and the right panel presents the wavenumber region 800–1300 cm^–1^: (a) pH 11, (b) pH 12.0, (c) pH 13.0.

The interfacial structural changes during the electrochemical oxidation of Ni(OH)_2_ and subsequent OER were studied by means of *in situ* SERS at different pH, keeping the ionic strength of the electrolyte constant; Fig. 2 shows the results obtained from these experiments.

The SERS spectra acquired at potentials below *ca.* 1.4 V *vs.* RHE show two weak peaks at 457 cm^–1^ and 504 cm^–1^ (see left panel of Fig. 2a–c), which can be assigned to the A_1g_ stretching modes of Ni–OH and Ni–O, respectively, in the Ni(OH)_2_.^35^–^37^ The stretching mode of the dehydrated form of nickel hydroxide (Ni–O peak at *ca.* 504 cm^–1^) has been attributed to a potential-assisted dehydration process of the nickel hydroxide to NiO-like structures, as expressed in eqn (2).^37^2
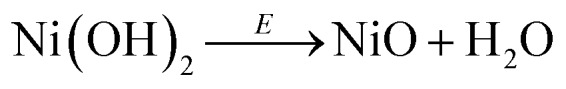



The Ni(OH)_2_/NiOOH redox transition occurs at potentials higher than *ca.* 1.35 V *vs.* RHE (see Fig. 1a), and the SERS spectra in the left panel of Fig. 2a–c show the appearance of two well-defined peaks at *ca.* 482 cm^–1^ and 562 cm^–1^ that can be assigned to the e_g_ bending vibration and the A_1g_ stretching vibration modes, respectively, of Ni–O in NiO(OH).^37^ The Raman peaks of Ni(OH)_2_ are weak in comparison with the intensity observed for the peaks assigned to NiO(OH), as previously reported by Bell's group; this has been attributed to the low Raman scattering cross-section of Ni(OH)_2_, in contrast to the stronger bands observed for NiO(OH) due to a resonance enhancing effect.^38^ At higher potentials, we observe the peak attributed to “active oxygen” in the oxyhydroxide structure in the 800–1150 cm^–1^ wavenumber region. The spectra in the right-hand panel of Fig. 2a–c show that the intensity of this peak increases as the pH of the electrolyte becomes more alkaline (spectra taken at pH 11.5 and 14 are shown in Fig. S4 in the ESI†).

**Scheme 1:**
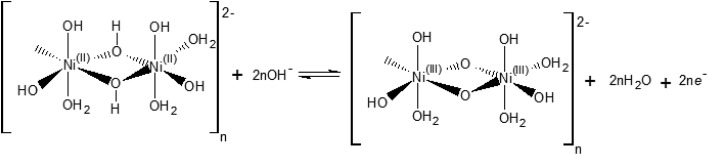
Electrochemical oxidation of a polymeric hydrous nickel(ii) hydroxide.

Our electrochemical results indicate that the oxidation of Ni(OH)_2_ occurs *via* a hydroxide-mediated deprotonation process that can be described by Scheme 1.

Scheme 1 shows the Ni^II^/Ni^III^ oxidation process of a polymeric hydrous nickel(ii) hydroxide ([(Ni^II^)_2_(OH)_6_(H_2_O)_3_]_*n*_^2–^), which is the actual state of the hydroxide on the electrode surface as reported by Lyons *et al.*^9^,^21^ This redox process is a OH^–^/e^–^ – coupled reaction which should exhibit no pH dependence on the RHE scale.

**Scheme 2:**
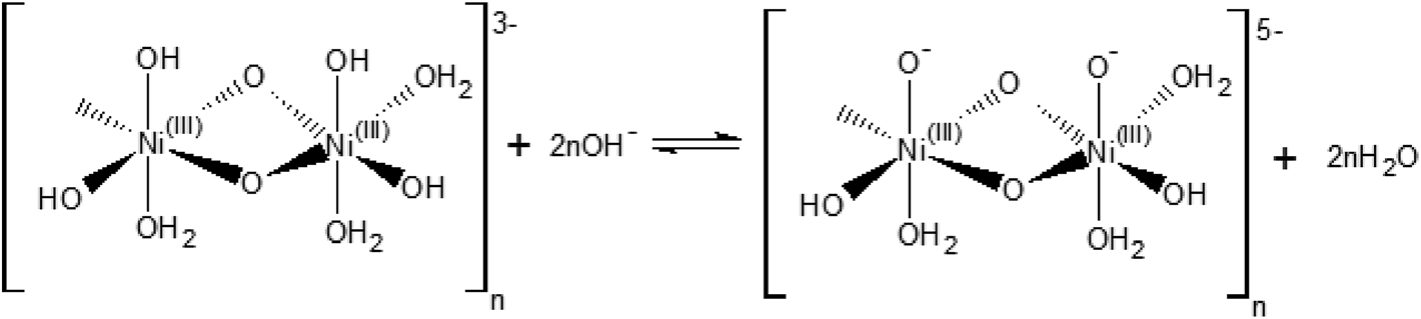
Hydroxide-mediated deprotonation process of the surface of NiO(OH) towards negatively charged species.

The [(Ni^III^)_2_O_2_(OH)_4_(H_2_O)_3_]_*n*_^2–^ may be further deprotonated if the pH of the electrolyte is higher than p*K*_a_ of the proton attached to the NiO(OH) species, leading to formation of a NiO^–^ species, as shown in Scheme 2.

The reaction presented in Scheme 2 is somewhat similar to the activation process towards oxygen evolution proposed by Nocera *et al.*^20^ for nickel-borate catalyst, which we have recently shown to be essentially identical to the NiOOH catalyst.^39^ The main difference between the deprotonation step depicted in Scheme 2 and the activation process of nickel-borate proposed by Nocera *et al.* is that Scheme 2 proposes a chemical deprotonation of the nickel oxyhydroxide towards a negatively-charged (or proton-deficient) surface species instead of a concerted proton – electron transfer step towards oxyl radicals; the generation of negatively charged surface species as shown in Scheme 2 allows to explain the strong pH dependency of the OER (see Fig. 1a and b).

We mention that we assume the oxidation state of nickel in NiO(OH) to be trivalent; this assumption is based on the XPS data indicating that the oxidation state of nickel in the oxyhydroxide is most likely trivalent.^40^–^43^

In a time-resolved spectroscopic study of photocatalytic water oxidation on Co_3_O_4_, Frei *et al.*^24^ also report a vibrational peak at *ca.* 1013 cm^–1^ when the photo-induced OER experiment was performed in H_2_^16^O. This peak shifts to lower frequencies (by *ca.* 47 cm^–1^) when the experiment is performed in H_2_^18^O. Based on the position of the peak in the spectrum and its shift in frequency due to the isotopic labeling, they assigned the vibrational peak to superoxo intermediates in the cobalt catalyst (CoOO). Moreover, the time-dependence of the peak intensity during the photocatalytic reaction led them to propose that the water oxidation occurs *via* decomposition of this superoxo species.

**Fig. 3:**
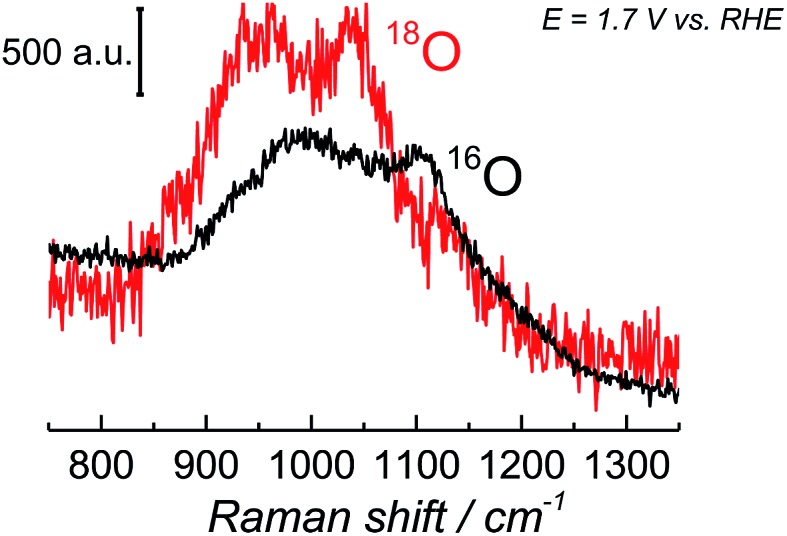
SER spectra of NiOOH in the wavenumber region 800–1350 cm^–1^. The spectra were obtained at 1.7 V *vs.* RHE in Na^16^OH 0.1 M. The electrolyte was prepared with H_2_^16^O and H_2_^18^O.

Since the frequency of the Raman peak attributed to the “active oxygen” species is very close to the position reported for the infrared peak assigned to the superoxo species on the cobalt-based catalyst,^24^ we performed a similar isotopic labeling experiment for the electrocatalytic oxygen evolution on NiOOH to confirm the nature of this species; Fig. 3 shows a comparison between the SER spectrum of the “active oxygen” in NiOOH, measured at pH 13 in H_2_^16^O and H_2_^18^O (at 1.7 V *vs.* RHE), showing a clear shift of the Raman peak to lower frequencies in the labeled media. The peaks attributed to the “active oxygen” shift *ca.* 64 cm^–1^ to lower frequencies in H_2_^18^O (see Table S1 in the ESI†). This value is close to the shift observed by Frei *et al.*^24^ for the superoxo species in cobalt oxide. The position of the “active oxygen” peak in the spectrum and its shift in H_2_^18^O therefore renders further credence to the assertion that the “active oxygen” peak corresponds to a superoxo (O–O) vibration (SERS of NiOOH at pH 13 in H_2_^18^O in the potential range of 1.45–1.75 V *vs.* RHE are shown in Fig. D5 in the ESI†). The nature of the shallow minimum in both spectra in Fig. 3 is unknown but suggests the existence of two spectroscopically discernible O–O species on the surface.

The superoxidic nature of the species in the SER spectra can be further confirmed by comparing the above SERS results to the existing DFT calculations of NiO_2_ complexes; the calculations show that NiO_2_ has vibrational modes in the wavenumber region 900–1150 cm^–1^,^44^ when the O_2_ in NiO_2_ is of peroxidic or superoxidic character, *i.e.* the 900–1150 cm^–1^ region corresponds to O–O stretching modes.

**Scheme 3:**
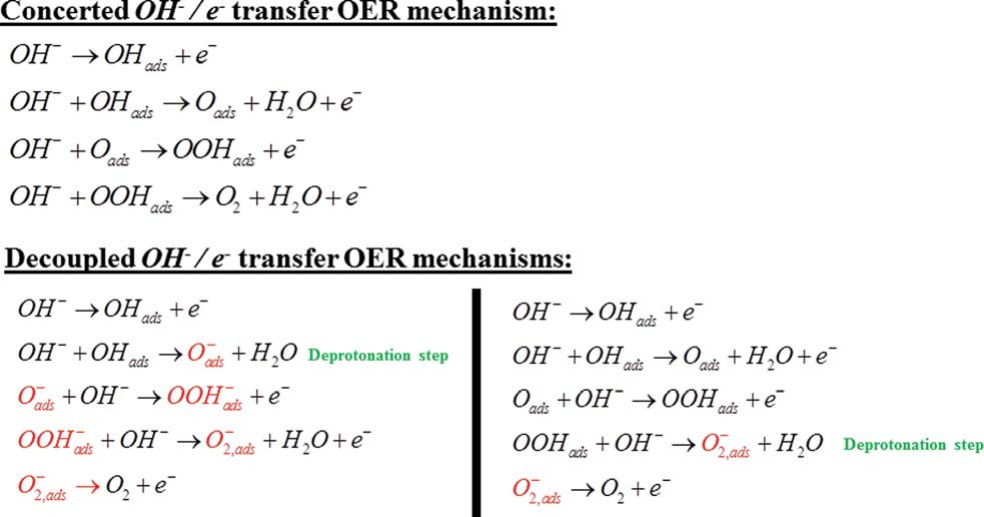
Comparison between the classical OER mechanism *via* concerted OH^–^/e^–^ transfer and the decoupled mechanisms *via* decomposition of the negatively charged superoxo (O_2_^–^) adsorbate generated by hydroxide-mediated deprotonation steps. The charges on the surface intermediates labeled in red should not be considered absolute, but as differential charges with respect to the surface species from which they are formed.

The dependence of the activity and the corresponding Raman bands on pH suggests that the species is formed upon deprotonation of the γ-NiOOH phase, which has been shown to be the more OER active phase of NiOOH.^26^ Moreover, in the light of the abovementioned results reported for the photocatalytic OER on cobalt oxide,^24^ the comparison to DFT calculations, the shift of the “active oxygen” Raman peak due to isotopic labeling and its pH-dependence, we conclude that this species is of superoxo nature and acts as precursor for oxygen evolution. As a consequence, we propose two possible mechanisms for the electrocatalytic OER on NiOOH that are similar to the one reported by Nocera *et al.*^20^ for the OER on nickel borate, and by Frei *et al.*^24^ for the light-assisted water oxidation on Co_3_O_4_. Our mechanisms incorporate a deprotonation step of the polymeric hydrous nickel oxyhydroxide towards formation of either oxide (NiO^–^) or superoxo species (NiOO^–^), as shown in Scheme 3. These mechanisms propose O_2_ formation *via* decomposition of the negatively charged surface species (O_2_^–^), which differs from the “classical” concerted OH^–^/e^–^ transfer mechanism that only considers uncharged adsorbates (or adsorbates with all equal charge).

The surface character of the pH-dependent active precursor (NiO^–^ or NiOO^–^) species is suggested by the Langmuir-type dependence of the OER activity on the NaOH concentration: a plot of 1/*j versus* 1/*C*_NaOH_ gives a reasonably straight line (see Fig. 1b). The deviation from linearity in the plot of Fig. 1b can be partially attributed to the strong pH dependence of the OER Tafel slope, as can be shown in Fig. 1c and d. The Tafel slope for OER on nickel oxyhydroxide varies from *ca.* 271 mV dec^–1^ at pH 11 to *ca.* 148 mV dec^–1^ at pH 13. Lyons *et al.*^9^ reported a similar trend in the values of the Tafel slope in experiments performed at higher concentrations of NaOH (0.1–5 M).

The decoupled OH^–^/e^–^ transfer mechanisms proposed in Scheme 3 consider two possible deprotonation pathways: OH_ads_ deprotonation towards negatively charged surface oxide (O_ads_^–^), or OOH_ads_ deprotonation towards negatively charged surface superoxide (O_2,ads_^–^); in both pathways the O_2_ formation occurs *via* decomposition of the negatively charged surface superoxide. The pH-dependence of the NiOOH activity towards OER may in principle be ascribed to both pathways: deprotonation giving rise to the negatively charged surface oxide (O_ads_^–^) and subsequent formation of the O–O bond, or OOH_ads_ deprotonation giving rise to the negatively charged surface superoxide. Ultimately, the (surface) p*K*_a_ of the corresponding acid–base equilibrium needs to be determined in order to assess which of the two explains the observed pH dependence. The importance of proton loss for generating localized reactive intermediates was also emphasized by Bediako *et al.*, who speculated that changes in ligand field strength upon deprotonation could localize the unpaired spin density to favor further reactivity.^20^

The relevance of negatively charged species, either of “O^–^” or of “OO^–^” character, on the surface of the catalyst during the OER has been suggested for many types of (transition-metal) oxide catalysts.^9^,^20^,^21^,^23^,^25^,^39^,^45^,^46^ However, theoretical descriptions of the OER mechanism employing density functional theory calculations have not yet incorporated this important pH effect in the reaction kinetics.^47^ We believe that the data reported here and the associated mechanism suggested in Scheme 3 provide another clear experimental example of the importance of negatively charged (surface) intermediates in generating pH dependent electrocatalytic activities, in agreement with a general model reported previously.^48^ Further understanding of the role of the oxo (MO^–^) and superoxo (MOO^–^) intermediates in the OER kinetics would require detailed DFT calculations that consider the p*K*_a_ and the relative stabilities of these intermediates. Future computational approaches towards modeling OER should account for this important acid–base surface chemistry.

## Conclusions

In this paper, we have provided spectro-electrochemical evidence for the active species that is responsible for the pH dependent OER activity of rigorously Fe-free NiOOH in alkaline electrolytes. We identify this species as a deprotonated γ-NiOOH surface phase in which stable (*i.e.* Raman observable) O–O bonds are formed. Based on our observations and other literature data on pH dependent OER kinetics, we propose a mechanism for the OER on NiOOH which is consistent with the observed pH-sensitivity; it involves the formation of a superoxo-type intermediate (NiOO^–^) that acts as preferential oxygen precursor at pH > 11. The proposed OER mechanism also considers the possibility of O_2_ formation *via* decomposition of OOH intermediates formed from the negatively charged surface oxide (NiO^–^). However, theoretical calculations to determine the (surface) p*K*_a_ of the corresponding acid–base equilibrium need to be performed to ultimately assess the relative importance of the NiOO^–^ and NiO^–^ intermediates in the OER mechanism. The pH dependence presented in this work rationalizes the unsuitability of NiOOH as electrocatalyst for applications in neutral or moderately alkaline pH (in the range 7–11), apart from the lower stability of the catalyst under these conditions.

## Supplementary Material

Supplementary information
