# Supplementary material for: The importance of nickel oxyhydroxide deprotonation on its activity towards electrochemical water oxidation
Source: Chem Sci. 2016 Jan 5;7(4):2639–45. doi: 10.1039/c5sc04486c (PMC5477031; doi:10.1039/c5sc04486c)
Supplement: Supplementary file 1 [file SC-007-C5SC04486C-s001.pdf]

Electronic Supplementary Material (ESI) for Chemical Science.
This journal is © The Royal Society of Chemistry 2016

SUPPORTING INFORMATION TO

The importance of nickel oxyhydroxide deprotonation on its activity towards electrochemical water oxidation

Oscar Diaz-Morales, David Ferrus-Suspedra, Marc T. M. Koper*

Leiden Institute of Chemistry, Leiden University, PO Box 9502, 2300 RA Leiden, The Netherlands.

Correspondence to: m.koper@lic.leidenuniv.nl

Table of Contents

The importance of nickel oxyhydroxide deprotonation on its activity towards electrochemical water oxidation S1

pH dependence of the potential for the Ni^{II}/Ni^{III} redox transition: comparison between Fe-free and Fe-containing electrolytes S2

Capacitance-corrected OER activity of NiOOH as a function of pH S3

The catalytic activity of NiOOH towards OER: the effect of iron impurities in the electrolyte S4

Additional SERS spectra of NiOOH in the Fe-free electrolyte S5

SERS experiments in solutions prepared with $H_2{}^{18}O$ S6

Position of the Raman peaks of NiOOH in electrolytes prepared with $H_2{}^{16}O$ and $H_2{}^{18}O$ S7

pH dependence of the potential for the Ni^{II}/Ni^{III} redox transition: comparison between Fe-free and Fe-containing electrolytes

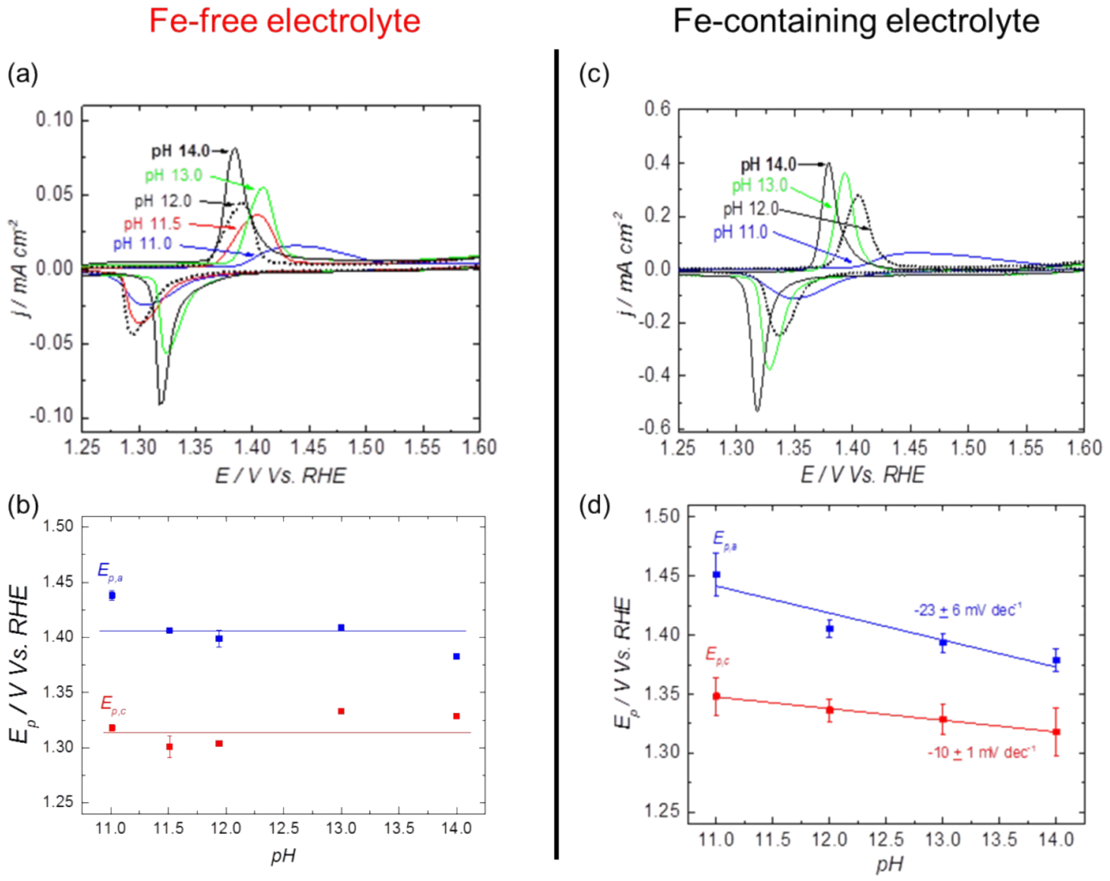

Figure S1. a) CVs of NiOOH acquired in purified (Fe-free) electrolyte, showing the changes in the Ni^{II}/ Ni^{III} redox transition with the pH. Scan rate: 0.01 V/s**.** b) Position of the oxidation and reduction peaks for the Ni^{II} / Ni^{III} redox transition ($E_{p,a}$ / $E_{p,c}$) in a) as a function of pH. c) CVs acquired in unpurified Fe-containing electrolyte, showing the changes in the Ni^{II}/ Ni^{III} redox transition with the pH. Scan rate: 0.01 V/s. d) Position of the oxidation and reduction peaks for the Ni^{II} / Ni^{III} redox transition ($E_{p,a}$ / $E_{p,c}$) in c) as a function of pH.

Capacitance-corrected OER activity of NiOOH as a function of pH

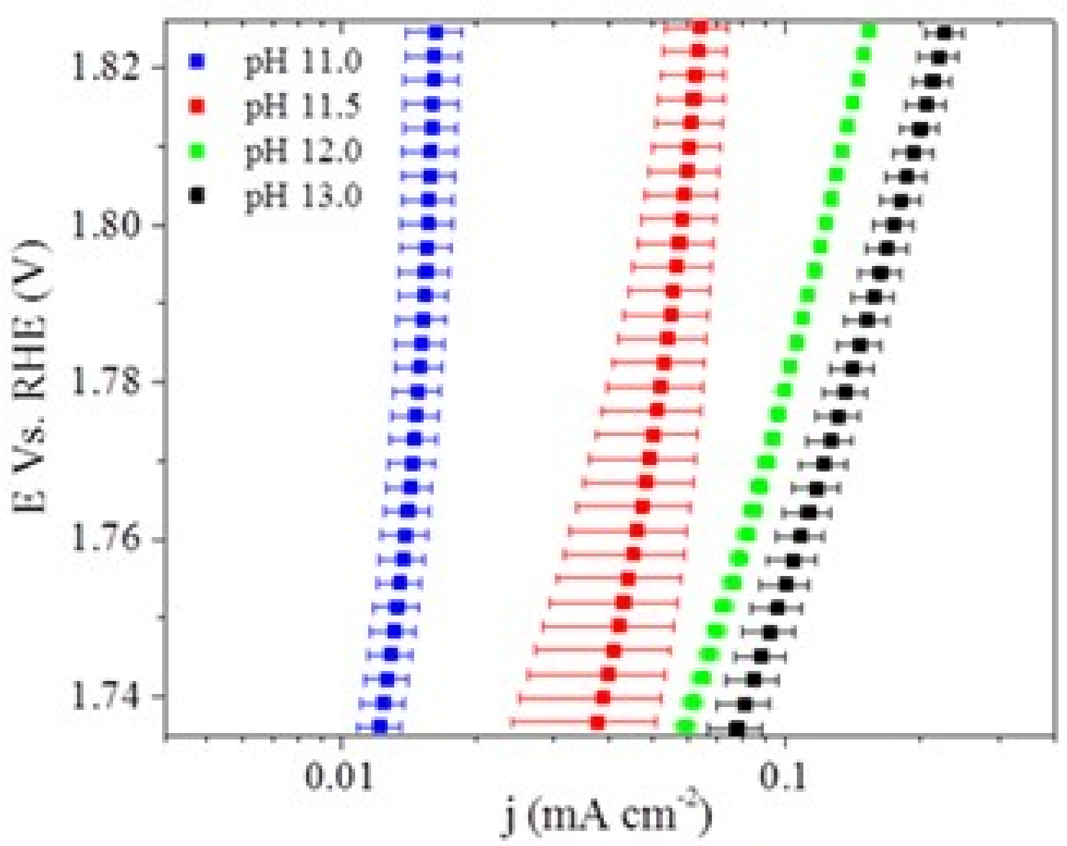

Figure S2. OER activity obtained from the average of the current measured in the forward and backward scan in the polarization curves of NiOOH deposited on Au (capacitance-corrected). Measurements at pH's 11 – 13 were performed at constant ionic strength, adjusted to 0.1 M with $NaClO_4$ except for pH 13, that solution was NaOH 0.1 M.

The catalytic activity of NiOOH towards OER: the effect of iron impurities in the electrolyte

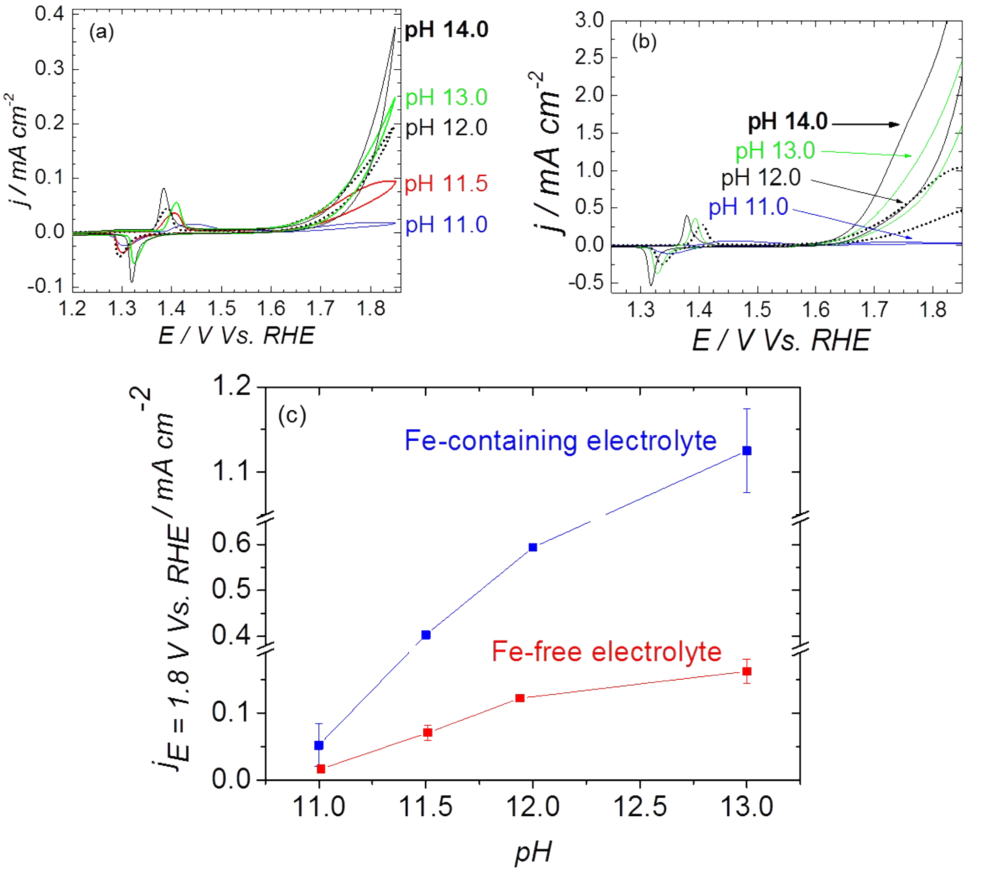

Figure S3. Effect of iron impurities on the activity of NiOOH towards OER. Measurements at pH's 11 – 13 were performed at constant ionic strength, adjusted to 0.1 M with $NaClO_4$. Solutions at pH 13 and pH 14 were NaOH 0.1 M and 1 M respectively. Scan rate: 0.01 V/s. a) CVs measured in Fe-free electrolyte. b) CVs measured in Fe-containing electrolyte. c) Capacitance-corrected activity of NiOOH towards OER as a function of pH, the activity was measured from the CVs a) and b) as the average of the backwards and forward current density at 1.8 V vs. RHE.

Additional SERS spectra of NiOOH in the Fe-free electrolyte

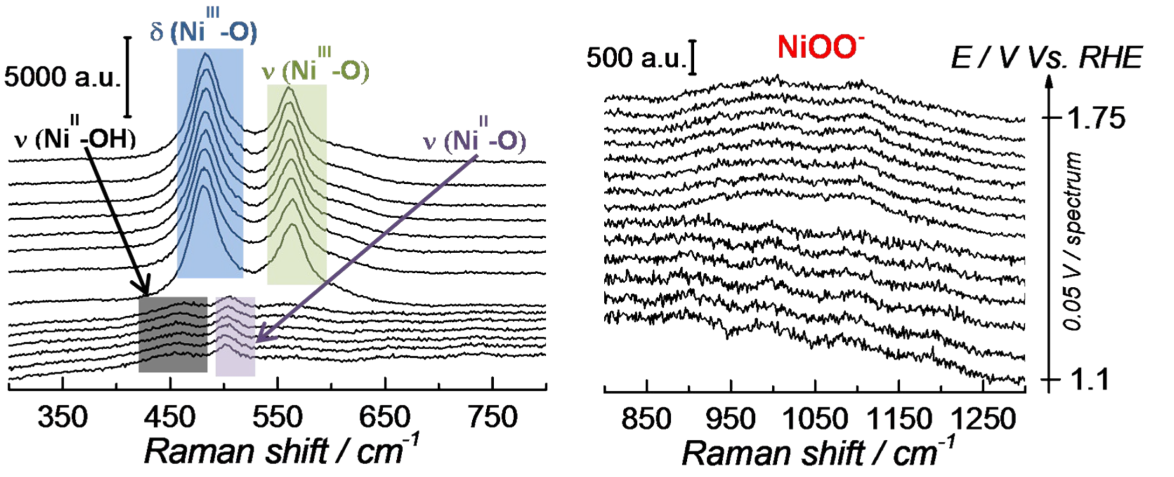

(b)

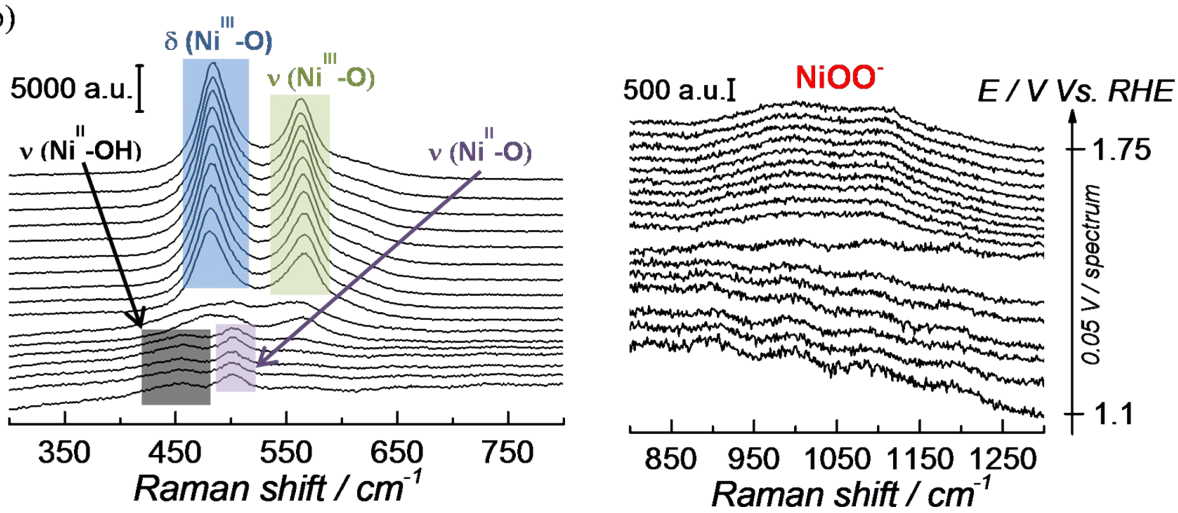

Figure S4. SER spectra obtained at constant potential during the electrochemical oxidation of $Ni(OH)_2$ and the subsequent OER on NiOOH at different pH's. The ionic strength of the solution was fixed to 0.1 M with $NaClO_4$. The left panel presents the spectra in the wavenumber region 300 – 800 cm^{-1} and the right panel presents the wavenumber region 800 – 1300 cm^{-1}: a) pH 11.5, b) pH 14.0.

SERS experiments in solutions prepared with $H_2{}^{18}O$

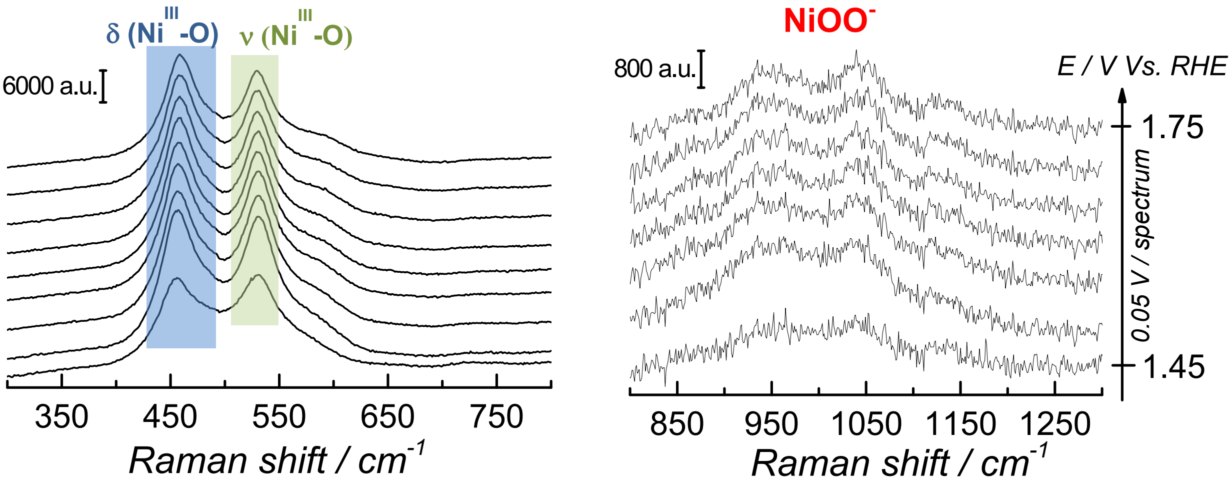

Figure S5. SER spectra obtained at constant potential during the electrochemical oxidation of $Ni(OH)_2$ and the subsequent OER on NiOOH at pH 13. The electrolyte was prepared in $H_2{}^{18}O$. The left panel presents the spectra in the wavenumber region 300 – 800 cm^{-1} and the right panel presents the wavenumber region 800 – 1300 cm^{-1}.

Position of the Raman peaks of NiOOH in electrolytes prepared with $H_2{}^{16}O$ and $H_2{}^{18}O$

Table S1. Position of the Raman peaks associated with the vibrations δ(Ni^{III}-O), ν(Ni^{III}-O) and $NiOO^-$ as a function of the oxygen isotope in the electrolyte. The spectra were obtained in NaOH 0.1 M in potentiostatic conditions.

E (V vs. RHE)	δ(Ni^{III}-O)		ν(Ni^{III}-O)		$NiOO^-$	
	^{16}O	^{18}O	^{16}O	^{18}O	^{16}O	^{18}O
1.65	481 ± 1	459 ± 3	561 ± 1	530 ± 5	1040 ± 10	981 ± 15
1.7	482 ± 1	460 ± 1	561 ± 1	531 ± 3	1044 ± 16	973 ± 10
1.75	481 ± 1	458 ± 3	560 ± 1	529 ± 5	1044 ± 15	982 ± 14
